# Prognostic factors in treatment of traumatic femoropopliteal arterial injuries at a Brazilian trauma center

**DOI:** 10.1590/1677-5449.202200202

**Published:** 2022-09-19

**Authors:** Gustavo Henrique Dumont Kleinsorge, Pedro Gustavo Rezende Teixeira, Claudia Caroline Barbosa Pfannes, Rodrigo Di Vita do Lago, Simone de Campos Vieira Abib

**Affiliations:** 1 Fundação Hospitalar do Estado de Minas Gerais – FHEMIG, Hospital João XXIII, Belo Horizonte, MG, Brasil.; 2 University of Texas, Austin Dell Medical School, Austin, TX, USA.; 3 Universidade Federal de São Paulo – UNIFESP, São Paulo, SP, Brasil.

**Keywords:** femoral artery, popliteal artery, injury, trauma, artéria femoral, artéria poplítea, lesão, trauma

## Abstract

**Background:**

Despite significant improvements in outcomes, traumatic arterial limb injuries remain a significant cause of limb loss and mortality.

**Objectives:**

This study sought to identify predictors of mortality and major amputation in patients undergoing revascularization after femoropopliteal arterial trauma.

**Methods:**

This was a retrospective review of a trauma registry from an urban trauma center in Brazil. All patients admitted to our hospital with a femoropopliteal arterial injury from November 2012 to December 2017 who underwent vascular reconstruction were included. Univariate analyses and logistic regression analyses were conducted to identify factors independently associated with the primary outcome of amputation and the secondary outcome of mortality.

**Results:**

Ninety-six patients were included. Eleven patients (11.5%) had an amputation and 14 (14.6%) died. In the logistic regression model for amputation, patients with ischemia duration greater than 6 hours were approximately 10 times more likely to undergo an amputation compared to those with ischemia duration less than or equal to 6 hours (adjusted odds ratio (AOR) [95% confidence interval (CI)]: 9.6 [1.2-79.9]). The logistic regression model for mortality revealed that patients with ischemia duration greater than 6 hours were approximately 6 times more likely to die compared to those with ischemia duration less than or equal to 6 hours (AOR [95% CI]: 5.6 [1.3 to 24.7).

**Conclusions:**

Ischemia duration remains the most important factor independently associated with limb loss and mortality for patients undergoing femoropopliteal arterial revascularization after traumatic injuries. Physiological status on admission and trauma scores are also important.

## Resumo

**Contexto:**

As lesões arteriais traumáticas de membros ainda permanecem uma causa significativa de perda de membros e mortalidade, apesar de melhorias significativas observadas nos resultados após a ocorrências dessas lesões.

**Objetivos:**

Este estudo buscou identificar preditores de mortalidade e amputações em pacientes submetidos à revascularização após trauma arterial femoropoplíteo.

**Métodos:**

Esta é uma revisão de um Registro de Trauma Vascular. Todos os pacientes com lesão arterial femoropoplítea internados em nosso hospital de novembro de 2012 a dezembro de 2017 e submetidos a reconstrução vascular foram incluídos. Análises univariadas, seguidas de análises de regressão logística, foram realizadas para identificar fatores independentemente associados com os resultados primários de amputação e mortalidade.

**Resultados:**

Foram incluídos 96 pacientes, com média de 27 anos. O *Revised Trauma Score* (RTS) foi, em média, 7,152; já o *Injury Severity Score* (ISS) foi, em média, 15. Onze pacientes (11,5%) tiveram amputação, e 14 pacientes (14,6%) morreram. Observou-se que pacientes com o tempo de isquemia maior que 6 horas apresentaram aproximadamente 10 vezes mais chance de amputação do que aqueles com tempo igual ou menor que 6 horas (intervalo de confiança de 95% [IC95%]: 1,2 a 79,9). O tempo de isquemia maior que 6 horas aumentou em aproximadamente 6 vezes a chance de mortalidade (IC95%: 1,26 a 24,77). A instabilidade hemodinâmica aumentou em 9 vezes a chance de mortalidade (IC95%: 2,36 a 36,67).

**Conclusões:**

O tempo de isquemia continua sendo o fator mais importante independentemente associado a amputação e óbito em pacientes submetidos à revascularização arterial femoropoplítea após traumas. O estado fisiológico e os escores de trauma são importantes.

## INTRODUCTION

Trauma is one of the main causes of death globally^1^ and vascular trauma accounts for 0.65% to 1.14% of cases.^2^ Despite their relative low incidence, vascular injuries are associated with potentially devastating complications and remain a challenge to the professionals who care for injured patients. Treatment of vascular lesions of the lower limbs has two main objectives: the first is to save the patient’s life, the second is to save the patient’s limbs.^3^ Despite improvements in outcomes since the second world war, vascular trauma involving the lower limbs is still associated with a high incidence of amputations.^4^ Treatment of these lesions has improved over time with the introduction of endovascular procedures, but there are still many controversies about the best approach.

Brazil is a very large country and has a highly heterogeneous healthcare system. Most Brazilian cities lack organized emergency care and the scarcity of resources is a significant challenge to definitive treatment of complex vascular lesions, requiring patients to be transferred to referral hospitals, resulting in significant treatment delays and potential negative impacts on outcomes. National best-practice guidelines for pre-hospital or intra-hospital management of these injuries are not available and institutions with protocolized care are the exception. A few publications have described the management of vascular trauma and their outcomes in Brazil,^5^^-^9 but no publications have specifically addressed management of femoropopliteal arterial trauma and its outcomes.

The aim of this study was to identify factors independently associated with major amputation and mortality in patients undergoing revascularization after femoropopliteal arterial trauma. Identification of these factors may suggest areas in which opportunities for quality improvement exist at both institutional and trauma system levels.

## METHODS

The study was approved by the Research Ethics Committee (CAAE: 27171219.8.0000.5505, protocol 3.976.718).

This was a retrospective review of a vascular trauma registry. This electronic institutional registry captures all patients with vascular injuries admitted to our hospital and is maintained by the vascular surgery team. Specific vascular data are entered by the attending surgeon involved with each case immediately after the surgical procedure. At the time of patient discharge, the document is completed with information regarding outcomes occurring during the in-hospital period. For the purposes of the present study, all patients undergoing femoropopliteal arterial vascular reconstruction after sustaining blunt or penetrating trauma from November 2012 to December 2017 were included.

Variables abstracted included patient demographics, mechanism of injury, evidence of ischemia on admission, vascular injury type (arterial transection, thrombosis or arteriovenous fistula), presence of associated peripheral nerve injury (intraoperative diagnosis), types of treatment (embolectomy, interposition of great saphenous vein, primary reconstruction, or temporary shunt), use of perioperative heparinization (local or systemic), presence of soft tissue lesions, and additional surgical procedures performed. The primary outcome was amputation and the secondary outcome was death, both within 30 days.

Statistical analysis was performed using R version 3.2.5, MINITAB, and SPSS version 18. Overall descriptive statistics were calculated for the study population. Univariate analyses were performed for both outcomes to identify factors independently associated with amputation and mortality. Categorical variables were analyzed using Pearson’s chi-square test or Fisher’s exact test (when expected frequencies were below 5) and continuous variables were analyzed using the Mann-Whitney test and Student’s *t* test as appropriate. Multivariate logistic regression models were then estimated including all factors associated with each of the outcomes from the univariate analyses based on clinical relevance and a p-value cutoff of 0.25. These models were used to identify independent associations between the various risk factors and amputation or death. P values < 0.05 were considered statistically significant.

This study is reported using the STROCSS guideline criteria.^10^

## RESULTS

**Figure 1:**
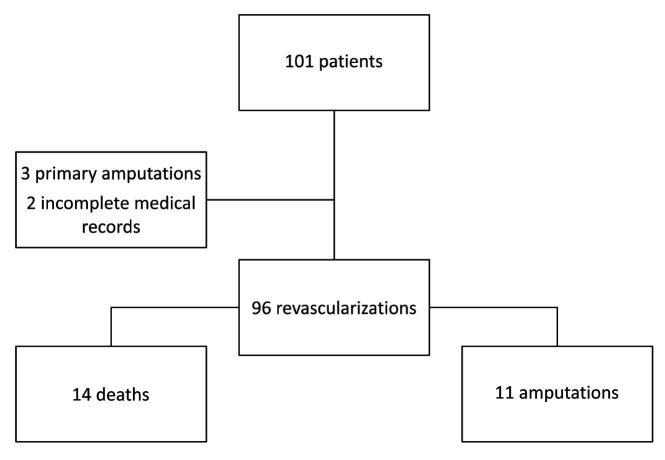
Flowchart illustrating results.

A total of 101 patients met the inclusion criteria during the 5-year study period. Three patients who needed primary amputation at admission and two patients with incomplete medical records data were excluded. Mean age was 27 years (range 8 to 62 years). Mean revised trauma score (RTS) was 7.152 (range 1.163 to 7.841) and mean injury severity score (ISS) was 15 (range 9 to 34). Additional characteristics are summarized in Table 1. The overall amputation rate was 11.5% (11 patients) and mortality was 14.6% (14 patients). All amputations and deaths occurred during the hospital stay (Figure 1).

**Table 1:** Population characteristics.

**Characteristics**	**Frequency**	
	**n**	**%**
Gender		
Female	9	9.4
Male	87	90.6
Ischemia duration		
≤ 6 hours	44	45.8
> 6 hours	52	54.2
Ischemia on admission		
Yes	86	89.6
No	10	10.4
Bone fracture		
Yes	49	51.0
No	47	49.0
Peripheral nerve disruption		
Yes	27	28.1
No	69	71.9
Soft tissue disruption		
Yes	54	56.3
No	42	43.7
Associated surgery		
Yes	60	62.5
No	36	37.5
Trauma mechanism		
Blunt	34	35.4
Penetrating	62	64.6
Hemodynamic stability		
Stable	68	70.8
Unstable	28	29.2
Injured artery		
CFA	4	4.2
SFA	59	61.4
Popliteal	33	34.4
Mechanism of vascular injury		
AVF	5	5.2
Transection	63	65.6
Thrombosis	28	29.2
Treatment		
Embolectomy	2	2.1
GSV interposition	76	79.1
Primary reconstruction	9	9.4
Temporary shunt	9	9.4
Perioperative heparinization		
Local	16	16.7
Systemic, 5000 UI	72	75.0
No	8	8.3
Fasciotomy		
Yes	54	56.3
No	42	43.7

CFA: common femoral artery; SFA: superficial femoral artery; AVF: arteriovenous fistula; GSV: great saphenous vein

Among the 11 patients undergoing amputation, with a mean age of 27 years, 10 (90.9%) were male; 10 (90.9%) had ischemia lasting more than 6 hours; all had evidence of ischemia on admission; the mechanism was blunt trauma in 6 (54.5%) patients and penetrating trauma in 5 (45.5%); 5 (45.5%) had an associated fracture, 5 (45.5%) had peripheral nerve injury, and 7 (63.6%) had soft tissue injury; 7 (63.6%) underwent associated surgeries; 4 (36.4%) presented with hemodynamic instability; 6 (54.5%) had superficial femoral artery (SFA) injury and 5 (45.5%) had popliteal artery injury; in 7 (63.6%) patients arterial section was the mechanism of vascular injury and 4 (36.4%) had thrombosis; 9 (81.8%) underwent treatment with great saphenous vein (GSV) interposition and 2 (18.2%) had temporary shunts; 2 (18.2%) had local perioperative heparinization, 6 (54.5%) had systemic heparinization, and 3 (27.3%) did not receive heparin; and 7 (63.4%) patients underwent fasciotomy (Table 2).

**Table 2:** Univariate analyses for amputation.

**Caracteristics**	**Amputation**			**p-value**
	**Yes**	**No**	**OR (CI_95%_)**	
Gender (male)	10 (90.9%)	77 (90.6%)	1.04 (0.1 to 50.7)	1.000^(2)^
Age (year)	27 ± 12.4 (20)	28 ± 9.3 (28)	-3.0 (-9.0 to 4.0)	0.438^(3)^
Trauma mechanism (penetrating)	5 (45.5%)	57 (67.1%)	0.4 (0.1 to 1.8)	**0.189** ^(2)^
Ischemia duration (> 6 hours)	10 (90.9%)	42 (49.4%)	10.2 (1.3 to 83.5)	**0.009** ^(1)^
Ischemia on admission	11 (100%)	75 (88.2%)	3.2 (0.2 to 66.9)*	0.598^(2)^
Hemodynamic status (unstable)	4 (36.4%)	24 (28.2%)	1.4 (0.3 to 6.3)	0.725^(2)^
RTS	6.041 ± 2.4 (7.841)	7.296 ± 1.3 (7.814)	0.0 (-2.7 to 0.001)	**0.133** ^(3)^
ISS	13 ± 3,7 (16)	16 ± 6.3 (16)	0.0 (-7.0 to 0.001)	**0.202** ^(3)^
Associated surgeries	7 (63.6%)	53 (62.4%)	1.1 (0.2 to 5.3)	1.000^(2)^
Injured artery				1.000^(2)^
CFA	0 (0.0%)	4 (4.7%)	1.0	
SFA	6 (54.5%)	53 (62.4%)	1.1 (0.04 to 31.7)^*^	
Popliteal	5 (45.5%)	28 (32.9%)	1.7 (0.1 to 51.8)*	
Types of vascular injury				0.856^(2)^
AVF	0 (0.0%)	5 (5.9%)	0.5 (0.02 to 13.9)	
Transection	7 (63.6%)	56 (65.9%)	0.7 (0.2 to 2.6)	
Thrombosis	4 (36.4%)	24 (28.2%)	1.0	
Treatment				0.471^(2)^
Embolectomy	0 (0.0%)	2 (2.4%)	0.0 (0.0 to 0.0)*	
GSV interposition	9 (81.8%)	67 (78.8%)	0.4 (0.1 to 2.2)*	
Primary reconstruction	0 (0.0%)	9 (10.6%)	0.2 (0.01 to 4.5)*	
Temporary shunt	2 (18.2%)	7 (8.2%)	1.0	
Perioperative heparinization				**0.056** ^(2)^
Local	2 (18.2%)	14 (16.5%)	0.3 (0.02 to 2.9)	
Systemic, 5000 UI	6 (54.5%)	66 (77.6%)	0.2 (0.02 to 1.3)	
No	3 (27.3%)	5 (5.9%)	1.0	
Fasciotomy	7 (63.4%)	47 (55.3%)	1.4 (0.3 to 7.1)	0.751^(2)^
Bone fracture	5 (45.5%)	44 (51.8%)	0.8 (0.2 to 2.7)	0.694^(1)^
Peripheral nerve disruption	5 (45.5%)	22 (25.9%)	2.4 (0.5 to 10.4)	0.282^(2)^
Soft tissue injury	7 (63.6%)	47 (55.3%)	1.4 (0.3 to 7.1)	0.751^(2)^
				

^(1)^Chi-square;

^(2)^Fisher’s exact;

^(3)^Mann-Whitney;

OR (CI95%) odds ratio, 95% confidence interval; RTS: Revised Trauma Score; ISS: Injury Severity Score; CFA: common femoral artery; SFA: superficial femoral artery; AVF: arteriovenous fistula; GSV: great saphenous vein;

* Calculated by regression model with penalized likelihood estimator method.

The logistic regression model for amputation showed that patients with ischemia duration greater than 6 hours were approximately 10 times more likely to undergo an amputation compared to those with ischemia duration less than or equal to 6 hours (adjusted odds ratio [AOR], 95% confidence interval [CI]: 9.6 [1.2-79.9]). With each unit increase in RTS, the likelihood of amputation reduced by 43% (95%CI: 2-96%) (Table 3).

**Table 3:** Adjusted odds ratios for amputation (95%CI).

	**OR**	**CI_95%_**		**p-value**
Ischemia duration				
> 6 hours	9.6	1.2-79.9		**0.036**
RTS	0.7	0.51-0.98		**0.037**

Variables included: Trauma mechanism (penetrating), Ischemia duration (> 6 hours), RTS, ISS, Perioperative heparinization.

The 14 deaths occurring in this series were secondary to brain injury in two cases, to complications from ischemia-reperfusion syndrome in five cases, to exsanguination in six cases (all six in extremis upon arrival), and to pulmonary embolism in one case.

All 14 of the patients who died were male, with a mean age of 25 years. Mean RTS and ISS were 4.559 and 20, respectively. Eleven patients (78.6%) had ischemia duration exceeding 6 hours; 13 (92.9%) had ischemia on admission. The trauma mechanism was blunt in 6 (42.9%) patients and penetrating in 8 (57.1%); 8 (57.1%) patients had associated fracture, 3 (21.4%) had peripheral nerve injury, and 11 (78.6%) had soft tissue disruption; 10 (71.4%) underwent associated surgeries; 10 (71.4%) presented hemodynamic instability on admission; 3 (21.4%) had common femoral artery (CFA) injury, 10 (71.4%) had SFA injury, and 1 (7.2%) had popliteal artery injury; in 9 (64.3%) the mechanism of vascular injury was artery transection and in 5 (35.7%) it was thrombosis; 1 (7.1%) underwent embolectomy, 6 (42.9%) had GSV interposition, and 7 (50.0%) had temporary shunt; 2 (14.3%) had local perioperative heparinization, 5 (35.7%) had systemic heparinization, and 7 (50.0%) did not receive heparin; and 7 (50.0%) patients underwent fasciotomy (Table 4).

**Table 4:** Univariate analyses for mortality.

**Characteristics**	**Mortality**			**p**
	**Yes**	**No**	**OR (CI_95%_)**	
Gender (male)	14 (100%)	73 (89%)	3.8 (0.2 to 79.3)*	0.348^(2)^
Age (year)	25 ± 7.8 (23)	29 ± 9.8 (28)	-4.0 (-9.0 to 2.0)	**0.223** ^(3)^
Trauma mechanism (penetrating)	8 (57.1%)	54 (65.8%)	0.7 (0.2 to 2.7)	0.556^(2)^
Ischemia duration (> 6 hours)	11 (78.6%)	41 (50.0%)	3.7 (0.95 to 14.1)	**0.047** ^(1)^
Ischemia on admission	13 (92.9%)	73 (89.0%)	1.6 (0.2 to 75.6)	1.000^(2)^
Hemodynamic status (unstable)	10 (71.4%)	18 (22.0%)	8.6 (2.2 to 42.3)	**<0.001** ^(2)^
RTS	4.559 ± 2.1 (4.621)	7. 549 ± 0.9 (7.841)	-2.8 (-4.5 to -1.9)	**<0.001** ^(3)^
ISS	20 ± 7.7 (16)	15 ± 5.5 (16)	5.1 (1.8 to 8.5)	**0.003** ^(4)^
Associated surgeries	10 (71.4%)	50 (61.0%)	1.6 (0.5 to 5.5)	0.455^(1)^
Injured artery				**0.002** ^(2)^
CFA	3 (21.4%)	1 (1.2%)	**1.0**	
SFA	10 (71.4%)	49 (59.8%)	0.1 (0.001 to 1.01)	
Popliteal	1 (7.2%)	32 (39.0%)	0.02 (0.002 to 0.3)	
Types of vascular injury				0.789^(2)^
AVF	0 (0.0%)	5 (6.1%)	0.4 (0.01 to 10.6)^*^	
Transection	9 (64.3%)	54 (65.9%)	0.7 (0.2 to 2.4)*	
Thrombosis	5 (35.7%)	23 (28.0%)	**1.0**	
Treatment				**<0.001** ^(2)^
Embolectomy	1 (7.1%)	1 (1.2%)	0.3 (0.01 to 7.8)*	
GSV interposition	6 (42.9%)	70 (85.4%)	0.03 (0.01 to 0.2)*	
Primary reconstruction	0 (0.0%)	9 (11%)	0.02 (0.001 to 0.5)*	
Temporary shunt	7 (50.0%)	2 (2.4%)	**1.0**	
Perioperative heparinization				**<0.001** ^(2)^
Local	2 (14.3%)	14 (17.1%)	0.03 (0.0004 to 0.3)	
Systemic 5000 UI	5 (35.7%)	67 (81.7%)	0.01 (0.0002 to 0.1)	
No	7 (50.0%)	1 (1.2%)	**1.0**	
Fasciotomy	7 (50.0%)	47 (57.3%)	0.7 (0.2 to 2.3)	0.610^(1)^
Bone fracture	8 (57.1%)	41 (50.0%)	1.3 (0.4 to 4.2)	0.621^(1)^
Peripheral nerve disruption	3 (21.4%)	24 (29.3%)	0.7 (0.1 to 2.8)	0.751^(2)^
Soft tissue injury	11 (78.6%)	43 (52.4%)	3.3 (0.9 to 12.8)	**0.069** ^(1)^

^(1)^Chi-square;

^(2)^Fisher’s exact;

^(3)^Mann-Whitney;

^(4)^Student’s *t*;

OR (CI95%) odds ratio, 95% confidence interval; RTS: Revised Trauma Score; ISS: Injury Severity Score; CFA: common femoral artery; SFA: superficial femoral artery; AVF: arteriovenous fistula; GSV: great saphenous vein;

* Calculated by regression model with penalized likelihood estimator method.

The logistic regression model for mortality revealed that patients with ischemia duration greater than 6 hours were approximately 6 times more likely to die compared to those with ischemia duration less than or equal to than 6 hours (AOR [95% CI]: 5.6 [1.3 to 24.7). Additional factors independently associated with mortality were hemodynamic instability and ISS. Hemodynamically unstable patients were approximately 9 times more likely to die (AOR [95% CI]: 9.3 [2.4-36.6]). For each unit increase in the ISS, the likelihood of death increased by 14% (95% CI: 3%-26%) (Table 5).

**Table 5:** Adjusted odds ratios for mortality (95%CI).

	OR	CI_95%_		P-value
Ischemia duratuin				
> 6 hours	5.6	1.26-24.77		**0.024**
Hemodynamic status				
Unstable	9.3	2.36-36.67		**0.001**
ISS	1.14	1.03-1.26		**0.014**

Variables included: Age (years), Ischemia duration (> 6 hours), Hemodynamic status (unstable), RTS, ISS, Injured artery, Treatment, Perioperative heparinization, Soft tissue injury.

## DISCUSSION

In the present study describing the experience of a Brazilian trauma center with the treatment of femoropopliteal arterial injuries over a 5-year period, ischemia duration greater than 6 hours and low RTS were found to be independently associated with amputation after revascularization. It was also demonstrated that prolonged ischemia duration, hemodynamic instability on admission, and elevated ISS were independently associated with mortality.

These findings are in accordance with a meta-analysis by Perkins et al.,^11^ in which ischemia duration exceeding 6 hours was associated with a fourfold increase in the risk of amputation. Alarhayem et al.^12^ demonstrated that longer time between trauma and the operating room was associated with a greater risk of amputation. These publications highlight the need for a trauma system that enables fast access to a hospital where resources are available for definitive treatment of such injuries. Amputation rates in patients who underwent repair within 1 hour were 6%, compared with 12% and 13% in those who underwent repair after 1 to 3 hours and 3 to 6 hours respectively. The present study demonstrated that with each unit increase in the RTS, the risk of amputation is reduced by 43%. Patients with low RTS have very poor physiological status. In these cases, prolonged surgical procedures, such as complex revascularization surgery, should be avoided because they increase the risk of morbidity and mortality. In a study published by Futchko et al.,^13^ it was shown that 20% of patients who underwent an amputation during hospitalization presented on admission with tachycardia and hypotension. Although this article did not directly measure the RTS value, it shows that poor physiological characteristics were significantly associated with amputation.

Our findings that ISS and hemodynamic instability are factors independently associated with mortality after vascular trauma are in agreement with a single-center retrospective cohort study published by Perkins et al.^14^ In our study, prolonged ischemia duration was associated with increased mortality, possibly due to complications related to ischemia-reperfusion syndrome. Circulation of metabolic products after revascularization may result in renal failure, pulmonary complications, heart failure, and disseminated intravascular coagulation. These conditions can significantly worsen the patient’s condition and their detrimental effect is proportional to muscle mass and the duration of ischemia.^15^ The present study highlights the elevated morbidity and mortality following vascular trauma. Patients with an ISS greater than 16 and who were admitted with hemodynamic instability had significantly higher mortality, which underscores the importance of careful judgment when complex revascularization procedures are being considered for these patients. Although some earlier studies have failed to demonstrate a relationship between ischemia duration and limb outcome,^16^ our results demonstrated that every effort should be made to shorten the duration of ischemia. The present study included polytrauma patients and not only those with isolated femoropopliteal vascular trauma, which may explain the higher mortality rates observed when compared to other series.^3^^,^^17^

The findings from this study suggest that aggressive hemorrhage control in the prehospital setting and reduction of ischemia duration are key points to avoid loss of limbs and life. Tourniquets are not widely used in our city and there is no protocol for use of this bleeding-control tool by pre-hospital care teams. Another peculiarity of our emergency care system is that some patients are transported to the trauma center by the police, due to urban violence, and law-enforcement personnel are not equipped with or trained to use tourniquets. This may explain why some patients arrived at the hospital with poor physiological status. One of the authors has previously demonstrated that use of tourniquets is associated with a sixfold reduction in mortality in patients with peripheral vascular injuries.^18^ Personnel involved in prehospital care should be trained in the Stop-the-Bleed protocol to avoid preventable hemorrhagic death.^19^ Our hospital is the regional referral center for many surrounding cities. Although some of these patients could potentially be managed locally, in practice most patients with traumatic vascular injuries are sent to our center. One potentially actionable item would be reorganization of the inter-hospital agreement protocols for rapid air transport of these patients after hemodynamic stabilization, which could prevent patients undergoing prolonged ischemia duration.

Temporary shunts are a tool that can potentially shorten the duration of ischemia. Glass et al.^20^ proposed an evidence-based algorithm in which revascularization using a vascular shunt must precede bone fixation. At our institution, use of temporary shunts was mostly reserved for damage control scenarios as we do not systematically use them to shorten the ischemia duration in cases in which there is an associated fracture. Our local practice reflects findings from a retrospective multicenter study by Inaba et al.,^21^ in which use of shunts was more frequent for damage control procedures compared to temporary use to shorten the ischemia duration in cases of associated fracture. This concept of “vessel first” when there is an associated bone fracture requiring surgical fixation is an area that warrants consideration at our institution.

This study has several limitations including its retrospective design, the relatively small number of participants, and its single-center nature. Also, the ischemia duration being recorded as a dichotomized variable (more or less than six hours) precluded a more detailed analysis of the implications of time-to-revascularization. Associated venous lesions were not evaluated, therefore it was not possible to establish whether their presence or absence had an impact in the outcomes.

## CONCLUSION

In summary, our findings suggest that ischemia duration remains the most important factor independently associated with loss of limb and life for patients undergoing femoropopliteal arterial revascularization after traumatic injuries. Physiological status on admission and trauma scores are also important prognostic factors in this setting. Local protocols to guide the best treatment for these patients must be established and the greatest efforts must be made to reduce the duration of ischemia before revascularization.
